# Intercontinental collaboration in clinical trials for children and adolescents with cancer—A systematic review by ACCELERATE

**DOI:** 10.1002/cam4.4356

**Published:** 2021-10-23

**Authors:** Teresa de Rojas, Andrew J. Pearson, Nicole Scobie, Leona Knox, Darshan Wariabharaj, Pamela Kearns, Gilles Vassal, Gregory Reaman, Todd Alonzo, Andrea Biondi, Kathy Brodeur‐Robb, Teresa de Rojas, Maryam Fouladi, Thomas Gross, Stephen Hunger, Pamela Kearns, Geoff McCowage, Alberto Pappo, Andrew J. Pearson, Martin Schrappe, Maria Grazia Valsecchi, Gilles Vassal, Brenda Weigel, Peter Wejbora, James Whitlock, Michel Zwaan, Vickie Buenger, Leona Knox, Donna Ludwinski, Nicole Scobie, Elly Barry, Kathleen Neville, Anjali Sharma, Darshan Wariabharaj, Dominik Karres, Gregory Reaman

**Affiliations:** ^1^ ACCELERATE Brussels Belgium; ^2^ Zoé4life Sullens Switzerland; ^3^ Solving Kids’ Cancer UK London UK; ^4^ Janssen Research & Development, LLC Raritan New Jersey USA; ^5^ Cancer Research UK Clinical Trials Unit National Institute for Health Research (NIHR) Birmingham Biomedical Research Centre Institute of Cancer and Genomic Sciences Birmingham UK; ^6^ Paediatric and Adolescent Oncology Department Gustave Roussy Cancer Campus INSERM U1015 Université Paris‐Saclay Villejuif France; ^7^ Food & Drug Administration Silver Spring Maryland USA

**Keywords:** adolescent cancer, childhood cancer, clinical research, clinical trials, drug development, international collaboration, rare diseases

## Abstract

**Background:**

Since pediatric cancer drug development is a global enterprise, we sought to provide an overview of the landscape of intercontinental clinical trials in pediatric oncology opened over the last decade.

**Methods:**

ClinicalTrials.gov was systematically searched to identify all clinical therapeutic trials which opened between 2010 and 2020 and recruited pediatric patients (<18 years) with cancer.

**Results:**

Over the last 10 years, 295 (8.7%) of 3383 therapeutic pediatric cancer trials were international and 182 (5.4%) were intercontinental. Most intercontinental trials were phase‐1 or 2, with 25% late‐phase, 65% were sponsored by industry, and North America was involved in 92%. Industry‐sponsored proportionally more phase‐1 trials than academia (41% vs. 25%); conversely, academia sponsored more phase‐2 and late‐phase trials (39% and 31% vs. 36% and 21%, respectively) (*p* = 0.020). North America–Europe collaboration was predominantly industry sponsored as opposed to North America–Oceania and Europe–Oceania collaboration, more frequently academic (*p* < 0.0001). Most late‐phase trials (18/20, 90%) focusing on pediatric malignancies were conducted by academic sponsors and 10 of these were conducted by Children's Oncology Group (COG)/National Cancer Institute in the United States and Oceania. There was no significant increase over time of intercontinental trials and a trend for a reduction in academic trials.

**Conclusions:**

Despite the relative rarity of childhood malignancies, especially within molecular subtypes, only 5.4% of pediatric cancer trials were intercontinental. The number of intercontinental trials remains small, with no significant increase over the last decade. The ACCELERATE International Collaboration Working Group aims to identify existing hurdles and propose solutions to improve intercontinental collaboration in clinical research for the benefit of children and adolescents with cancer.

## Graphical abstract

Intercontinental collaboration is alarmingly rare in childhood cancer trials, despite the rare disease setting and despite pediatric clinical research being inevitably a global enterprise. Barriers to collaboration should be identified and met with specific solutions to accelerate urgently needed drug development for children and adolescents with cancer.

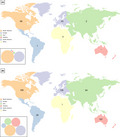

## 1 INTRODUCTION

The survival of childhood cancer has improved significantly over the last five decades, with recent reported overall 5‐year survival rates of 79%–85% in children (<14 years) and 82%–85% in adolescents (15–19 years).^1^, ^2^, ^3^ This improvement has been achieved due to collaborative, multidisciplinary practice‐changing academia‐led clinical trials and improvements in clinical care. However, survival rates for all pediatric malignancies have plateaued over the last decade, and for certain malignancies remain unacceptably low. Therefore, innovative treatments with new mechanisms of action are urgently needed.^4^, ^5^, ^6^ Moreover, a lack of new effective, but less toxic drugs results in long‐term morbidities in most survivors.

The era of personalized medicine has brought an encouraging, yet complex landscape for cancer care.^7^ New molecular subtypes are emerging, creating very small subgroups of patients. For example, four new molecular entities of central nervous system embryonal tumors, have been recently described, converting the very rare disease group formerly called primitive neuroectodermal tumors) into even smaller groups of patients.^8^ In view of the relative rarity of childhood malignancies, and especially when considering molecular subtypes, international trials between countries in the same continent and intercontinental collaboration are key for conducting science‐driven and evidence‐generating clinical trials. Moreover, the widest possible choice of therapeutic options should be provided to as many patients with relapsed disease and their families as feasible. Although, globally, all stakeholders are working closely together to accelerate drug development for children and adolescents,^9^ many hurdles, including operational, regulatory, and financial challenges remain to implement intercontinental, wide‐reaching collaborative trials. While there have been some notable examples of success such as the Children's Oncology Group (COG)‐EsPhALL phase 2 trial of dasatinib and chemotherapy in newly diagnosed Philadelphia chromosome‐positive acute lymphoblastic leukemia (ALL) (CA180‐372),^10^ or the EURAMOS‐1 phase 3 trial investigating intensified postoperative chemotherapy in patients with a poor response to preoperative chemotherapy for newly diagnosed high‐grade osteosarcoma,^11^ many other intercontinental trials have been very slow to open.

ACCELERATE is a multi‐stakeholder international platform, created to accelerate drug development through innovation for children and adolescents with cancer,^9^ with patient advocates, academic clinicians and researchers, representatives of biotechnology and biopharmaceutical companies, and regulators as equal partners. The International Collaboration Working Group was constituted following the 2019 ACCELERATE Annual Conference, with the purpose of improving international collaboration in childhood cancer clinical research by identifying obstacles and challenges and proposing potential solutions.^12^ This manuscript is the first output of the Group to better define the landscape of international collaboration and focuses specifically on intercontinental trials.

We conducted a systematic review of clinical trials in pediatric oncology opened over the last decade to determine: (1) the number of trials which were conducted intercontinentally and (2) the type and focus of intercontinental collaboration trials in childhood cancer.

## 2 METHODS

### 2.1 Trial selection

The ClinicalTrials.gov database^13^ was searched to identify all clinical trials (early and late phase, including post‐marketing authorization studies) which opened between 2010 and 2020 and recruited pediatric patients (<18 years) with cancer. The ClinicalTrials.gov database was selected as it is the largest publicly available trial database and includes the majority of registered trials.

The search was performed in October 2020 with the term “Cancer” (all fields) and filtered by “Child (birth‐17)” (Age Group) AND “Interventional (Clinical Trial)” (Study Type) AND “from 01/01/2010 to 01/01/2020” (Start Date).

All studies focusing on cancer and meeting the search criteria were reviewed. Only international trials (open at sites in ≥2 countries) were included. The exclusion criteria were applied hierarchically: (1) Not international (i.e., conducted in only one country), (2) Non‐cancer‐directed trials, (3) Typically adult malignancies (prostate, gynecological, gastrointestinal, bladder, and lung), (4) Follow‐up/rollover studies, and (5) Trials investigating psychosocial or behavioral interventions (e.g., exercise modification).

Supportive care and diagnostic procedure trials that were specifically cancer‐directed were included, as they constitute an essential part of cancer care.

A review of ClinicalTrials.gov was performed by one investigator (T.d.R.) and eligibility of all identified studies was assessed by at least two investigators (T.d.R./G.R.). Data extraction was performed by one investigator (T.d.R.) with quality review by three investigators (G.R./A.P./G.V.). In case of discrepancy, the decision was taken by consensus of the investigators. Further data cleaning/validation was carried out by T.d.R.

### 2.2 Data extraction

Variables were partially extracted automatically from the ClinicalTrials.gov database and were thereafter reviewed and manually encoded through a standardized data extraction form (Microsoft Excel 2016).

The collected variables per trial included (1) General variables: the National Clinical Trial number, title, sponsor, status, location, availability of results (publication), start and completion date (actual or projected); (2) Study design variables: phase, masking, randomization, sample size; and (3) Study population and objectives variables: condition (tumor types included in each trial), age, experimental interventions (single and combination), and primary outcome.

### 2.3 Definition of trial variables

The sponsor was considered academia for trials sponsored by universities, hospitals and/or academic research institutions/groups, or industry for trials sponsored by pharmaceutical companies. The sponsor classification by clinicaltrials.gov was followed. COG/National Cancer Institute (NCI) trials were grouped together as a subcategory of academic trials.

Location was categorized as North America, South America, Europe, Asia, Oceania, Africa, or intercontinental (for those with two or more participating continents). Intercontinental trials were subcategorized according to the involved continents (e.g., North America–Europe, Europe–Asia‐Oceania, etc.).

Trials were considered phase 1 (including phase 1/2), phase 2, late phase (phase 2/3 or phase 3).

Primary outcome measures were considered as efficacy (when measuring response rate, incidence of adverse events (AEs) for support interventions, technique effectiveness, etc.), survival (overall survival, event‐free survival, etc.), safety (incidence of AEs, dose‐limiting toxicities, maximum tolerated dose, etc.), feasibility, quality of life (including patient‐reported outcomes), quality of care (including care planning), or as biomarker evaluation (including pharmacokinetics, area under the curve, etc.).

The definitions of other trial variables are shown in Data S1.

### 2.4 Statistical analysis

The median and interquartile range (IQR) were used to describe quantitative data. Percentages were used to describe qualitative data. Percentages may not always total 100% due to rounding error. The χ^2^ or the Fisher exact test were used for statistical comparisons when appropriate. Multiple linear regression models were used to analyze evolution in the number of trials over time (one for each analysis). The R software v.3.4.0 was used to perform data processing and data analysis, and to plot the results. The maps were created using the free online software mapchart.^14^

The study methodology complies with the Preferred Reporting Items for Systematic Reviews and Meta‐Analyses (PRISMA) statement and guidelines whenever applicable to the registry meta‐research context.^15^

## 3 RESULTS

**FIGURE 1:**
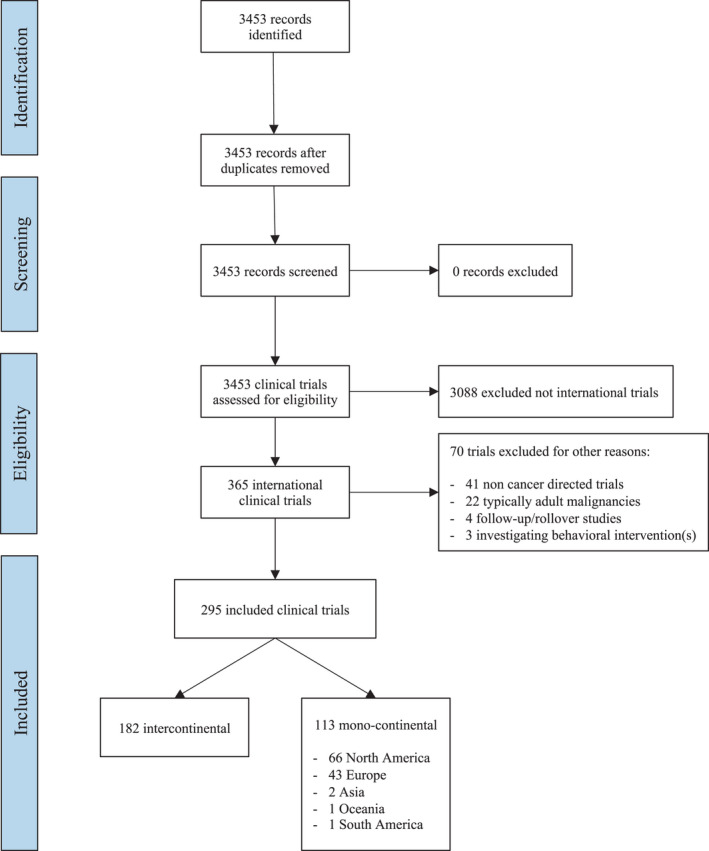
PRISMA flow diagram showing the number of clinical trials identified and the eligibility process. PRISMA, Preferred Reporting Items for Systematic Reviews and Meta‐Analyses

**FIGURE 2:**
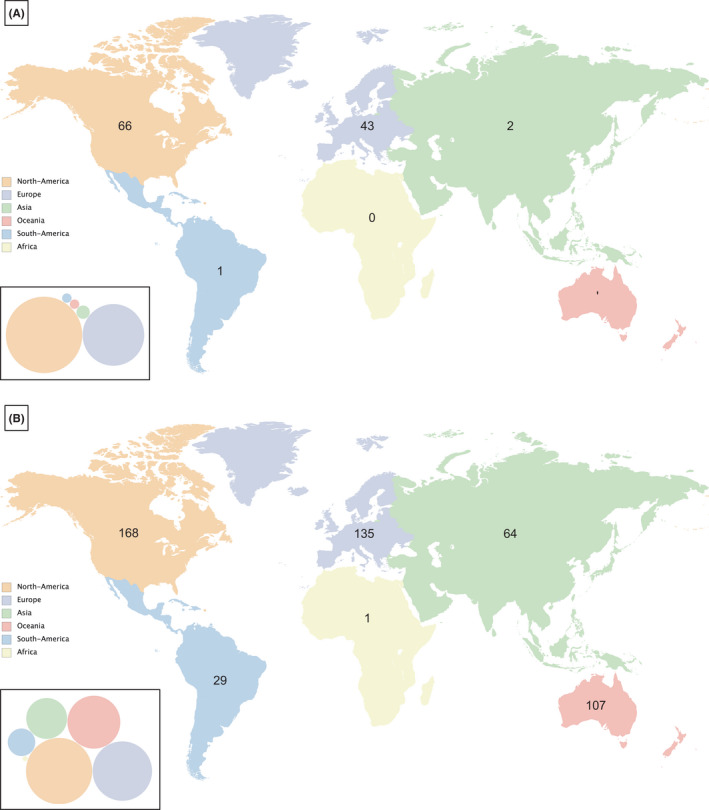
Location of international trials for (A) mono‐continental trials (*n* = 113); and (B) intercontinental trials (*n* = 182). The size of the bubbles shown in the bubble chart at the bottom‐left of each panel is proportional to the number of trials per continent. Of note: Central America has been considered as part of South America for the purposes of this study

Three thousand four hundred and fifty‐three trials were identified (Figure 1); 70 trials of these were not therapeutic trials targeting a pediatric malignancy (41 non‐cancer directed, 22 related to predominantly adult malignancies, 4 follow‐up/rollover studies; and 3 behavioral interventions). Two hundred and ninety‐five of the remaining 3383 (8.7%) were international trials, and 182 (5.4%) were intercontinental. Trials conducted exclusively in one continent accounted for 38% (113/295) and most of these were in North America (66/113) or Europe (43) (Figure 2).

### 3.1 Intercontinental trials

Complete data were available for 182 intercontinental trials for the primary endpoint (location) and for most variables; unknown data were found for the variable “status” in ≤2% of the trials. The main characteristics of these trials are shown in Tables 1 and 2 and Figure 2B.

**TABLE 1:** Study design characteristics of the pediatric, intercontinental trials according to the sponsor

Study design	Total	Academic	Industry	*p*‐value
Number of trials	182	64	118	‐
Age				0.0003
Exclusively pediatric/AYA	105 (58%)	49 (77%)	56 (47%)	
Mixed^a^	77 (42%)	15 (23%)	62 (53%)	
Phase				0.020^b^
Phase 1	64 (35%)	16 (25%)	48 (41%)	
Phase 2	68 (37%)	25 (39%)	43 (36%)	
Late phase	45 (25%)	20 (31%)	25 (21%)	
Phase 4	2 (1%)	0	2 (2%)	
Not applicable^c^	3 (2%)	3 (5%)	0	
Randomization				0.007
Single arm	96 (53%)	27 (42%)	69 (58%)	
Multiple arms, not randomized	30 (16%)	8 (13%)	22 (19%)	
Randomized	56 (31%)	29 (45%)	27 (23%)	
Masking				1.000^b^
Open label	169 (93%)	60 (94%)	109 (92%)	
Blinded	13 (7%)	4 (6%)	9 (8%)	
Primary outcome				0.001^b^
Safety	74 (41%)	17 (27%)	57 (48%)	
Efficacy	50 (27%)	15 (23%)	35 (30%)	
Survival	47 (26%)	27 (42%)	20 (17%)	
Biomarker	8 (4%)	3 (5%)	5 (4%)	
Feasibility	3 (2%)	2 (3%)	1 (1%)	
Sample size among closed trials (*N* = 55): median (IQR)				‐
Phase 1 (*N* = 23)	36 (23–63)	16 (15–23)	49 (33–67)	
Phase 2 (*N* = 25)	30 (17–67)	75 (41–84)	25 (14–53)	
Late phase (*N* = 7)	433 (200–529)	200 (187–213)	480 (433–579)	

Note

Percentages may not always total 100% due to rounding error.

Abbreviations: AYA, adolescents and young adults; IQR, interquartile range.

^a^ Inclusion with upper age limit >40 years.

^b^ Chi‐squared test or Fisher Exact test was used whenever appropriate.

^c^ “Phase not applicable” trials consisted of a molecular platform trial, a pilot trial, and an imaging trial.

**TABLE 2:** Main characteristics of the intercontinental trials according to the sponsor

Global characteristics	Total	Academic	Industry	*p*‐value
Number of trials	182	64	118	‐
Continents involved				<0.0001
North America–Europe ± others	121 (66%)	16 (25%)	105 (89%)	
North America–Europe	44	8	36	
North America–Europe–Asia	16	1	15	
North America–Oceania–Europe	26	4	22	
North America–Oceania–Europe–Asia	9	0	9	
North America‐South America–Europe	1	0	1	
North America–South America–Europe–Asia	6	0	6	
North America–South America–Oceania–Europe–Africa	1	0	1	
North America–South America–Oceania–Europe–Asia	18	3	15	
North America–Oceania ± Others	43 (24%)	37 (58%)	6 (5%)	
North America–Oceania	37	32	5	
North America–Oceania–Asia	5	5	0	
North America–South America–Oceania	1	0	1	
Oceania–Europe ± Others	10 (5%)	9 (14%)	1 (1%)	
Oceania–Europe	7	7	0	
Oceania–Europe–Asia	1	1	0	
Oceania–Europe–South America	1	1	0	
Oceania–Europe–South America–Asia	1	0	1	
Other combinations	8 (4%)	2 (3%)	6 (5%)	
Europe–Asia	4	0	4	
North America–Asia	4	2	2	
Status				0.092
Ongoing^a^	124 (68%)	50 (78%)	74 (63%)	
Closed	55 (30%)	13 (20%)	42 (36%)	
Withdrawn	0	0	0	
Unknown status	3 (2%)	1 (2%)	2 (2%)	
Condition				0.781
Solid tumors	103 (57%)	34 (53%)	69 (58%)	
Hematologic malignancies	70 (38%)	27 (42%)	43 (36%)	
Mixed	9 (5%)	3 (5%)	6 (5%)	
Intervention				<0.0001
Single anti‐cancer medication	90 (49%)	6 (9%)	84 (71%)	
Targeted therapies	46	4	42	
Advanced therapies	17	0	17	
Immunotherapy	18	1	17	
Chemotherapy	9	1	8	
Combination	80 (44%)	48 (75%)	32 (27%)	
Novel–Classic	44	29	15	
Novel–Novel	20	4	16	
Classic–Classic	16	15	1	
Support therapy	7 (4%)	5 (8%)	2 (2%)	
Hematopoietic stem cell transplantation	2 (1%)	2 (3%)	0	
Diagnostic procedures	2 (1%)	2 (3%)	0	
Radiotherapy	0	0	0	
Surgery	1 (1%)	1 (2%)	0	
Published results among closed trials (*N* = 55)	40/55	9/13	31/42	‐

Note

Percentages may not always total 100% due to rounding error.

Fisher Exact Test was used for all calculations.

^a^ Of the 124 trials classified as “ongoing,” only one trial had the status “not yet recruiting.”

**FIGURE 3:**
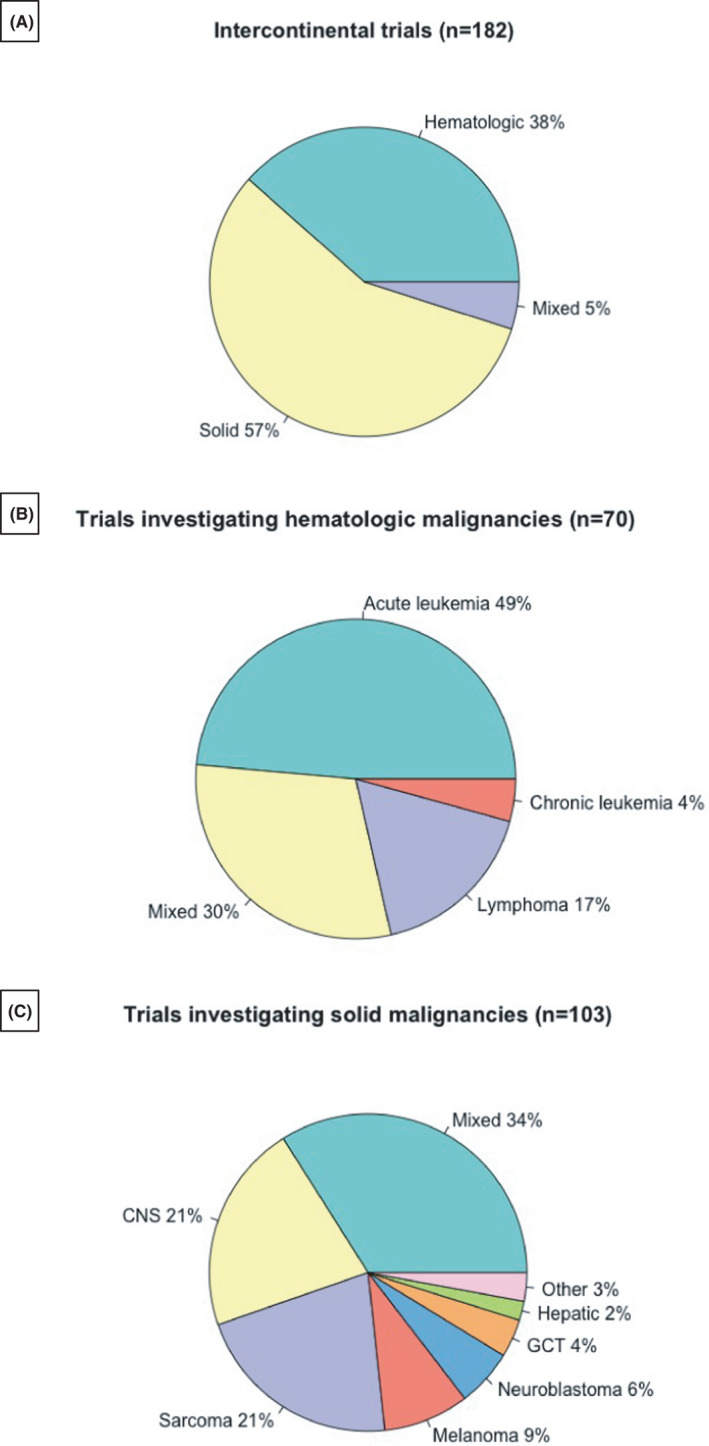
Proportion of intercontinental trials investigating different types of malignancies. (A) Overview of all trials; (B) Hematologic malignancies; (C) Solid malignancies. CNS, central nervous system; GCT, germ cell tumors

Sixty‐four (35%) were phase 1 trials, 68 (37%) phase 2 trials, and only 45 (25%) late‐phase trials (with two phase 4 trials and three “not applicable”). One hundred and twenty‐four (68%) trials were ongoing and 55 (30%) were closed (3 unknown status). One hundred and three (57%) investigated solid tumors and 70 (38%), hematological malignancies; nine trials (5%) investigated both (Figure 3).

#### 3.1.1 Sponsorship of intercontinental trials

In total, 118 of the 182 (65%) intercontinental trials were sponsored by industry and 64 (35%) were academically sponsored. Industry sponsored proportionally more phase 1 trials than academia (41% vs. 25%); conversely, academia sponsored more phase 2 (39% vs. 36%) and late‐phase trials (31% vs. 21%, respectively) (*p* = 0.020). Academic trials were more frequently randomized than industry trials (45% vs. 23%, *p* = 0.007), and had survival as the primary outcome with a higher proportion (42% vs. 17%, *p* = 0.001). Fifty‐eight percent of industry trials investigated solid tumors compared to 53% of academic trials (*p* = 0.781). Academia sponsored exclusively pediatric/adolescent and young adult (AYA) trials more frequently than the industry (77% vs. 47%, *p* = 0.0003).

#### 3.1.2 Geographical distribution of intercontinental trials

North America was involved in 168/182 (92%) of intercontinental trials, Europe in 135 (74%), Oceania in 107 (59%), Asia in 64 (35%), South America in 29 (16%), and Africa in one (0.5%). The most frequent intercontinental collaboration was between North America and Europe and/or other continents (121/182, 66%), followed by North America and Oceania and/or others (43, 24%). Europe and Oceania and/or others (without North America) collaborated in 10 (5%), and other combinations in eight (4%) trials. The North America‐Europe collaboration was predominantly industry sponsored as opposed to the North America‐Oceania and the Europe‐Oceania collaborations, which were more frequently driven by academia (*p* < 0.0001). The academic North America‐Oceania collaboration consisted mostly of trials run by COG/NCI (22/32, 69%).

The majority of industry‐sponsored phase 1 intercontinental trials, 43 (90%), involved both North America and Europe; 21 (44%) involved Oceania, 17 (35%) Asia, and only 4 (8%) involved South America. Similarly, industry‐sponsored phase 2 trials involved North America and Europe in 40 of 43 (93%) trials, Oceania in 14 (33%), Asia in 20 (47%), and South America in 9 (21%).

Eighteen of the 20 (90%) academically sponsored late‐phase trials focused on a malignancy predominantly occurring in children, for example, neuroblastoma (rather than melanoma), in contrast to 14 of 25 (56%) industry‐sponsored trials. Ten of 20 academically late‐phase trials sponsored intercontinental trials were run by COG/NCI; all included Oceania and five Asia, but only one included Europe and South America. Fourteen academic‐sponsored trials involved North America, 13 Europe, 14 Oceania, 9 Asia, and 4 South America. Only three trials involved all five continents and two North America–Oceania and Europe.

#### 3.1.3 Evolution over time

**FIGURE 4:**
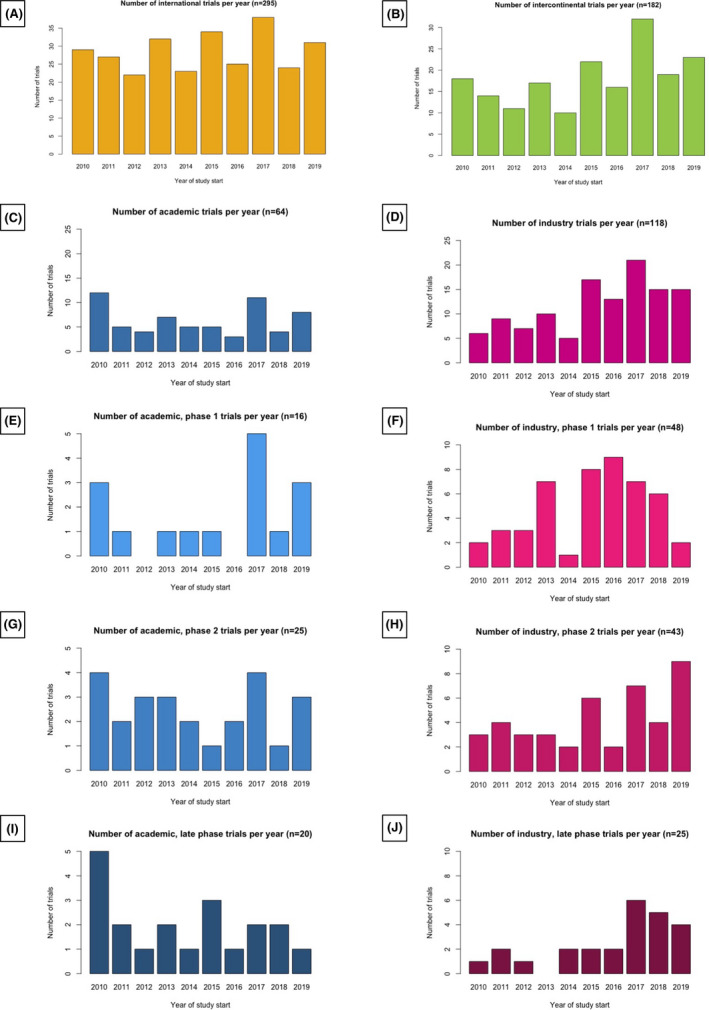
Evolution over time in the number of trials for (A) all international trials (including intercontinental and mono‐continental trials); (B) all intercontinental trials; (C) intercontinental trials with the academic sponsor; (D) intercontinental trials with industry sponsor; (E–J) intercontinental trials by sponsor and phase

There was no significant increase in the number of trials over time (Figure 4A); the first 5 years of the study (2010–2014) there were 70 intercontinental trials compared to 112 in the last 5 years (2015–2019) (*p* = 0.09; Figure 4B). There was a significant increase in industry trials from 37 to 81 (*p* = 0.01; Figure 4D), as opposed to the number of academic trials, which were reduced from 33 to 31 (*p* = 0.74; Figure 4C). The evolution over time in the number of intercontinental trials by sponsor and phase is shown in Figure 4E–J (Table S1).

#### 3.1.4 Other characteristics of intercontinental trials

Single anti‐cancer medications were investigated in 90/182 (49%) trials, with targeted therapies being most frequently explored 46/90 (51%). Combination trials were slightly less frequent (80/182, 44%). Industry trials investigated single medications in a significantly higher proportion than academic trials (71% vs. 9%); conversely, academic trials investigated combination therapies more frequently (75% vs. 27%) (*p* < 0.0001). In academic trials, investigated combinations involved most frequently novel and classic therapies (29/48, 60%), followed by the combination of different classic therapies (15, 31%). In contrast, industry trials investigated predominantly the combination of novel therapies (16/32, 50%) or of novel with classic therapies (15, 47%). Only seven (4%) trials investigated support therapy.

Ninety‐six trials were single‐arm (53%) and 169 (93%) open‐label. Thirty‐four of 45 (76%) late‐phase trials were randomized. The primary outcome was safety in 74/182 (41%), efficacy 50 (27%), and survival 47 (26%) trials. The median duration of the closed trials (*n* = 55) was 3.6 years (IQR 2.2–4.7). For phase 1 closed trials (*n* = 23), the median duration was 3.9 years (IQR 2.4–4.6); for phase 2 trials (*n* = 25), it was 2.8 years (IQR 1.8–4.7); and for late‐phase trials (*n* = 7), it was 4.3 years (IQR 3.5–5.1).

## 4 DISCUSSION

In view of the relative rarity of childhood cancer generally, and especially when considering molecular subtypes, international collaboration is essential to conduct biology‐driven, evidence‐generating, and practice‐changing clinical trials. Even for the more frequent malignancies, only intercontinental trials will allow pivotal questions to be answered and evaluation of promising agents to occur more rapidly. There is a need for intercontinental collaboration in pediatric oncology to design and conduct robust trials with sufficient sample size in a timely fashion to make meaningful conclusions to result in approval of new drugs and ultimately change practice. Also, providing access to new therapeutic options is very important for families of children with relapsed disease, as it is important that they believe ‘no stone left unturned’ is achieved. Families seek hope through access to clinical trials, sometimes taking on significant burdens including extensive travel, large financial costs, and long periods of hospitalization and separation at a time when they may need the most intense support. This is exacerbated when there is progress (or perceived progress) elsewhere. Parent communities on social media form an interconnected world, resulting in increased awareness of therapeutic choices, with significant potential for peer influence in decision making. It is therefore imperative that the widest possible choice of therapeutic options are provided to as many children and their families (as close to home) as possible.

Although pediatric oncology is often held as a model of a collaborative philosophy and international collaboration,^16^ only 5.4% of trials being intercontinental suggests that there are substantial challenges in terms of intercontinental collaboration in clinical research, with no evidence of improvement over the last 10 years. In fact, only 182 intercontinental trials were conducted over 10 years, with almost two‐thirds (65%) being industry sponsored. The majority of intercontinental trials were phase 1 or phase 2 trials and only 25% were late phase.

We also observed substantial and statistically significant differences between intercontinental trials according to the sponsor in study design (age, phase, randomization, and primary outcome), investigated interventions, and location and continents involved. While academia‐sponsored phase 2 and late‐phase trials in a significantly higher proportion than the industry, industry sponsored more frequently phase 1 trials (*p* < 0.05).

The majority (75%) of phase 1 intercontinental trials were industry sponsored and involved North America and Europe in 95% of trials, Oceania in 31%, Asia in 49%, and South America in 23%. Industry‐sponsored phase 1 and phase 2 trials are more likely to involve Europe and predominantly investigate single agents as opposed to academic trials, which investigate combination therapies more frequently.

Most of the late phase trials (62%) focusing on malignancies predominantly occurring in children (e.g., hepatoblastoma) were carried out by academic sponsors and 10 of these trials were run by COG/NCI in the United States and Oceania. There is geographical disparity, with only one of these trials involving Europe. In contrast, most of the industry‐sponsored late‐phase trials focused on malignancies present in both adults and children (e.g., melanoma).

The distribution of academic‐sponsored intercontinental trials broadly reflected the distribution of childhood malignancies, however, trials sponsored by industry predominantly investigated solid tumors (58%).

This analysis highlighted that, despite the clearly stated need, there is a paucity of intercontinental trials, no significant increase over the last decade and a trend for a reduction in the number of academically sponsored trials. Early phase industry‐sponsored trials tended to involve Europe and North America, however, academic‐sponsored trials tend to be between North America and Oceania, many of which are sponsored by COG. The relative paucity of academically sponsored Europe and North America trials is striking. The overall predominance of industry‐sponsored trials may reflect the challenges for intercontinental trials conducted by academia in Europe and North America. Moreover, trial efficiency could be enhanced by including other continents such as Oceania. International collaboration in cancer research is highest among countries in close geographical proximity.^17^

Global inequality in pediatric cancer research is evidenced by our results, with large differences in the number of trials across continents. The paucity of identified trials in Africa and South America is remarkable. Nonetheless, intercontinental collaboration seems to facilitate research development in less economically and/or geographically favored continents, as seen by the number of mono‐continental versus intercontinental trials in South America (1 vs. 29, respectively) and Australia (1 vs. 107, respectively). This is not observed however in Africa, with multifaceted possible reasons having been pointed out by a recent work by SIOP Africa.^18^ Increasing intercontinental collaboration will hopefully contribute to reduce global inequality in cancer research and care.

There are multiple assumed hurdles to intercontinental collaboration in clinical trials—operational, regulatory, financial, and scientific. The Food and Drug Administration and the European Medicines Agency are working to facilitate international collaboration through discussions at Cluster Calls and other initiatives, aiming to improve international regulatory alignment. Furthermore, they are strongly encouraging simultaneous engagement across the Atlantic with regulators by sponsors.^19^

This systematic analysis of intercontinental clinical trials in childhood cancer is the first step toward identifying obstacles and proposing solutions, which will be addressed by the ACCELERATE International Collaboration Working group. It is not only paramount to enable broad access to trials, but another important aspect is to determine which trials need to be conducted in an intercontinental venue for timely efficient accrual—this will be addressed by the Working Group.

Despite the difficulties, intercontinental trials are feasible, as demonstrated by industry‐sponsored early phase trials and academic collaborative trials in North America and Oceania. In the COG‐EsPhALL phase 2 trial of dasatinib and chemotherapy in pediatric patients with newly diagnosed Philadelphia chromosome‐positive acute lymphoblastic leukemia (CA180‐372), North America, Europe, and Oceania collaborated successfully.^10^ The academic international, randomized, phase 3 trial involving Europe, North America, Oceania, and Asia for high‐risk, mature B‐cell non‐Hodgkin's lymphoma demonstrated the benefits of adding rituximab to standard chemotherapy.^20^ The industry‐driven, phase 1/2 SCOUT trial, which involved the same four continents, investigated the use of larotrectinib in tumors with NTRK‐fusion in children, and contributed to its histology‐agnostic approval.^21^, ^22^ Finally, the academic, randomized, phase 3 EURAMOS‐1 trial involving Europe, North America, and Oceania investigated intensified postoperative chemotherapy in newly diagnosed osteosarcoma, in patients with a poor response to preoperative chemotherapy, and stablished standard of care for this population.^11^

We acknowledge the limitations of this study. Clinicaltrials.gov was the only source used for the search, a registry that is being increasingly used by investigators to assess research practices. Conducting valid analyses requires an understanding of both the capabilities and limitations of the database.^23^ A major issue to be considered is that requirements for reporting trials have changed over time. However, the search for this study was limited to trials starting from 2010, notably after systematic registration of clinical trials was promoted in 2005.^24^ Nonetheless, the changing nature of clinicaltrials.gov needs to be considered when interpreting the results of the study, especially regarding the analysis of evolution over time, as well as our dependence on some of the categorizations given by the platform that might be misleading or incomplete (e.g., age, primary completion date). Another limiting aspect is the possible underrepresentation of trials conducted in low‐ and middle‐income countries, which may be listed in other registries,^23^, ^25^ or of small European trials only registered in EudraCT.

Despite these shortfalls, clinicaltrials.gov remains the largest publicly available trial database and included the majority of registered trials. Using MedLine, Embase or other literature databases would have limited the search to only trials with published results (58 of 295, 20%). Finally, we acknowledge that analyzing data from pediatric and AYA trials jointly may limit the conclusions. Nonetheless, since we are aiming to identify barriers and at a later stage propose solutions to increase intercontinental collaboration, we believe that a high‐level approach including pediatric and AYA issues is appropriate given the global nature of our work. A more detailed AYA‐specific analysis is currently under preparation, in collaboration with the ACCELERATE FAIR (Fostering Age Inclusive Research) working group.

In summary, it appeared that, establishing academically sponsored phase 1 trials and academically sponsored trials between North America and Europe was particularly challenging. In contrast, academically sponsored trials between North America and Oceania, many of which are sponsored by COG, seemed to be less difficult to implement.

In conclusion, the number of intercontinental trials in pediatric patients with cancer is not increasing, despite the acknowledged unmet medical need for international collaboration for rare pediatric tumor subtypes. Existing hurdles need to be defined and overcome to improve intercontinental collaboration in clinical research for the benefit of children and adolescents with cancer.

## ETHICAL CONSIDERATIONS

Ethical approval was not sought from an institutional review board nor ethics committee as it is not needed/applicable for this kind of study (systematic review with no inclusion of human subjects).

## CONFLICT OF INTEREST

The authors declare no conflict of interest.

## Supporting information

Supplementary Material

Supplementary Material

## Data Availability

All data collected for the study are publicly available at www.clinicaltrials.gov. A data dictionary is described in Section 2 (in the main text and additionally in Data S1). A database with the study trials and their main characteristics is provided as Data S2.
