# Supplementary material for: Intercontinental collaboration in clinical trials for children and adolescents with cancer—A systematic review by ACCELERATE
Source: Cancer Med. 2021 Oct 23;10(23):8462–74. doi: 10.1002/cam4.4356 (PMC8633236; doi:10.1002/cam4.4356)
Supplement: Supplementary file 1 — Supplementary Material [file CAM4-10-8462-s002.docx]

**Supplementary Material**

**Index**

Supplementary Methods………………………………………………………….Page 2

Supplementary Table 1…………………………………………………………...Page 5

**Supplementary Methods: Definition of trial variables (cont.)**

- **Status**: The status of the trials was categorized as ongoing (for those recruiting, enrolling by invitation, active not recruiting, not yet recruiting, or suspended), closed (for those completed -the study has ended normally, and participants are no longer being examined/treated- or terminated), and withdrawn (before enrolling its first patient).
- **Age**: The trials were classified in two main age groups as “exclusively pediatric/AYA (adolescents and young adults)” when the inclusion upper age limit was ≤40 years, and as “mixed” when the inclusion upper age limit was >40 years, and hence included older adults. This limit was chosen as internationally accepted to encompass children, adolescents and young adults.
- **Phase**: Trials were considered phase 1 (if early phase 1, phase 1, or phase 1/2), phase 2, late phase (if phase 2/3 or phase 3), or phase 4.
- **Sample size:** Sample size was only considered for closed trials.
- **Study design**: Trials were classified according to their design as single arm, multiple arms not randomized, or randomized. Masking was open-label or blinded.
- **Condition**: Investigated conditions were classified as solid tumors, hematological malignancies or mixed (when including both). Solid tumors included CNS tumors, sarcoma, neuroblastoma, melanoma and other skin tumors, germ cell tumors, hepatic tumors, gastro-intestinal tumors (GIST), nasopharyngeal tumors, and thyroid cancer. Solid tumors were considered mixed when including two or more different solid tumors. Hematological malignancies included acute leukemia, chronic leukemia, and lymphomas. They were considered mixed when including two or more different hematological malignancies.
- **Interventions**: The experimental interventions were those interventions being tested in the trials, as opposed to reference or control interventions. They were categorized as single anti-cancer medications, hematopoietic stem cell transplantation, surgery, radiotherapy, diagnostic procedures, and support therapy (for those interventions aimed at symptom control, infection prophylaxis, etc.). The anti-cancer medications were subcategorized as chemotherapies, molecular targeted therapies, immunotherapies (including checkpoint inhibitors, cytokines and vaccines), and advanced therapy medicines (including cell therapies and viruses).
  - Combination trials were those investigating more than one intervention. These were considered novel-novel when combining molecular targeted therapies with/or immunotherapies with/or advanced therapy medicines; classic-classic when combining any other types of interventions (e.g. chemotherapy with radiotherapy); and novel-classic when combining targeted therapies, immunotherapies or advanced therapies with any other intervention.
- **Dates**: The start date was defined as the actual date on which the first participant was enrolled in a trial, if applicable. For withdrawn or not yet recruiting trials, the estimated start date was used. For closed trials (i.e., completed or terminated), the completion date was defined as the date on which the last participant was examined or received an intervention to collect final data for the primary outcome measure(s). For ongoing or withdrawn trials, the estimated completion date was used. The duration of a closed trial was calculated from the start date to the completion date.

**Supplementary Table 1.** Linear regression models for time evolution

| Group of trials | N | β coefficient | P value of β | R^2^ | Fig. 4 panel |
| --- | --- | --- | --- | --- | --- |
| All trials (including international and intercontinental trials) | | | | | |
| International and intercontinental | 295 | 0.41 | 0.51 | 0.06 | A |
| Only intercontinental trials | | | | | |
| Total | 182 | 1.17 | 0.09 | 0.30 | B |
| Academic | 64 | -0.12 | 0.74 | 0.01 | C |
| Industry | 118 | 1.29 | **0.01** | 0.56 | D |
| Academic, phase 1 | 16 | 0.13 | 0.47 | 0.06 | E |
| Industry, phase 1 | 48 | 0.32 | 0.33 | 0.11 | F |
| Academic, phase 2 | 25 | -0.09 | 0.47 | 0.06 | G |
| Industry, phase 2 | 43 | 0.45 | 0.06 | 0.35 | H |
| Academic, late phase | 20 | -0.19 | 0.17 | 0.22 | I |
| Industry, late phase | 25 | 0.47 | **0.01** | 0.58 | J |

Significant P values are highlighted in bold letters.
